# Supplementary material for: Empirical methods for controlling false positives and estimating confidence in ChIP-Seq peaks
Source: BMC Bioinformatics. 2008 Dec 5;9:523. doi: 10.1186/1471-2105-9-523 (PMC2628906; doi:10.1186/1471-2105-9-523)
Supplement: Additional File 1 — A variety of html documents from the USeq web site detailing the available applications, their best usage, output file type descriptions, command line menus, etc. [file 1471-2105-9-523-S1.zip › USeqUserGuides/JavaDoc.zip!/JavaDoc/util/bio/digest/REDigest.html]

REDigest


|  |  |  |
| --- | --- | --- |
| **Overview** \| **Package** \| **Class** \| **Use** \| **Tree** \| **Deprecated** \| **Index** \| **Help** | |  |
| **PREV CLASS**   NEXT CLASS | **FRAMES**    **NO FRAMES**     **All Classes** |  |
| SUMMARY: NESTED \| FIELD \| CONSTR \| METHOD | DETAIL: FIELD \| CONSTR \| METHOD |  |


---


## util.bio.digest Class REDigest

```
java.lang.Object
  util.bio.digest.REDigest
```

---

``` public class REDigest extends java.lang.Object ```

StandAlone class to perform a restriction digestion on a sequence, a hack, uses as a template.

---

**Constructor Summary**

`REDigest()`


| **Method Summary** | |
| --- | --- |
| `static java.lang.String` | `getRestrictionMapString(java.lang.String seq, Enzyme[] enzymes)` |
| `static void` | `main(java.lang.String[] args)` |
| `static Enzyme[]` | `restrictionMap(java.lang.String DNA, Enzyme[] e)`             Runs thru an enzyme array attempting to cut a particular sequence |
| `static java.util.ArrayList` | `restrictionMapHTML(java.lang.String DNA, Enzyme[] e)`             Returns an arraylist of enzymes found to cut the sequence |
| `static java.lang.String` | `restrictionMapList(java.lang.String DNA, Enzyme[] e)`             Restriction map a sequence. |

**Methods inherited from class java.lang.Object**

`equals, getClass, hashCode, notify, notifyAll, toString, wait, wait, wait`

**Constructor Detail**

### REDigest

```
public REDigest()
```


**Method Detail**

### main

```
public static void main(java.lang.String[] args)
```

---


### restrictionMapHTML

```
public static java.util.ArrayList restrictionMapHTML(java.lang.String DNA,
                                                     Enzyme[] e)
```

:   Returns an arraylist of enzymes found to cut the sequence

---


### getRestrictionMapString

```
public static java.lang.String getRestrictionMapString(java.lang.String seq,
                                                       Enzyme[] enzymes)
```

---


### restrictionMap

```
public static Enzyme[] restrictionMap(java.lang.String DNA,
                                      Enzyme[] e)
```

:   Runs thru an enzyme array attempting to cut a particular sequence

---


### restrictionMapList

```
public static java.lang.String restrictionMapList(java.lang.String DNA,
                                                  Enzyme[] e)
```

:   Restriction map a sequence. Makes an ordered list of enzymes. First by number of cuts, second alphabetically.


---


|  |  |  |
| --- | --- | --- |
| **Overview** \| **Package** \| **Class** \| **Use** \| **Tree** \| **Deprecated** \| **Index** \| **Help** | |  |
| **PREV CLASS**   NEXT CLASS | **FRAMES**    **NO FRAMES**     **All Classes** |  |
| SUMMARY: NESTED \| FIELD \| CONSTR \| METHOD | DETAIL: FIELD \| CONSTR \| METHOD |  |


---
